# Absolute Stability Criteria for Large-Scale Lurie Direct Control Systems with Time-Varying Coefficients

**DOI:** 10.1155/2014/631604

**Published:** 2014-04-27

**Authors:** Fucheng Liao, Di Wang

**Affiliations:** ^1^School of Mathematics and Physics, University of Science and Technology Beijing, Beijing 100083, China; ^2^School of Automation and Electrical Engineering, University of Science and Technology Beijing, Beijing 100083, China

## Abstract

The absolute stability of large-scale Lurie direct control systems with time-varying coefficients is investigated. Based on the decomposition method for large-scale systems and technique of the nonsingular *M*-matrix, a suitable scalar Lyapunov function as a weighted sum is constructed. By estimating its total time derivative, some absolute stability criteria and practical corollaries are derived. Furthermore, the results are extended to multiple nonlinearities. The salient feature of this paper is that the criteria which we propose allow for the situation that the norms of time-varying coefficients are unbounded. The main idea of the methodology is that even if the coefficients are norm-unbounded, by restricting their relative magnitude, the problem of negative definiteness for the derivative can also be changed into the problem of stability for a constant matrix. Finally, some numerical examples are included to illustrate the effectiveness of the proposed criteria.

## 1. Introduction

The absolute stability problem has an important position in the analysis and design of control systems. In fact, as a typical class of nonlinear system, the problem of the absolute stability of the Lurie control system has been studied for almost 70 years [1–3] and has proved a fruitful area of research [4–8]. Recently, the problem of Lurie control systems has been extended. Among the studies, [9, 10] studied the absolute stability of large-scale Lurie systems, [11–13] considered the robust stability of uncertain Lurie systems, and [14, 15] discussed Lurie systems with time delays. In addition, there are many monographs on these topics [16, 17].

Nevertheless, most of the papers on Lurie control systems are confined to the norm-bounded coefficients. The absolute stability of Lurie control systems with time-varying and norm-unbounded coefficients has received little attention. In [18], a research method was introduced for the stability of large-scale systems with time-varying coefficients. The core result was that if the order of infinities for the interconnected elements is far less than the order of isolated subsystems, then the large-scale system is still asymptotically stable. Reference [19] promoted the results and considered the problem of robust exponential control for a class of large-scale systems with uncertainties and unbounded coefficients. On the other hand, the approach is also an effective way to investigate the Lurie control systems with time-varying coefficients. In particular, in the case of the Lurie indirect control systems, it is relatively easier to study them using this method. In [20, 21], this approach was applied to the Lurie indirect control systems with norm-unbounded coefficients, and some absolute stability criteria for this kind of system were obtained. Reference [22] subsequently extended the criteria to systems with multiple nonlinearities and large-scale Lurie indirect systems. However, because of the linear relationship between *σ* (*σ* is not an independent variable) and the other state variables in Lurie direct control systems, this became the main roadblock in judging the total time derivative of the Lyapunov function. Reference [23] overcame this difficulty and derived the sufficient condition of absolute stability for the Lurie direct control systems with norm-unbounded coefficients.

The problem proposed in this paper is more general than that described above: it focuses on large-scale Lurie direct control systems with time-varying coefficients and systems with multiple nonlinearities. The study of stability for large-scale systems is not a new one; it began in the 1960s [24], and the research method is basically the decomposition method for large-scale systems [25, 26]. From the viewpoint of cybernetics, this is known as decentralized control [27, 28]. The basic idea is decomposing the large-scale system into a certain number of lower-order isolated subsystems and constructing the Lyapunov function of the large-scale system through the isolated subsystems. Our objective in the following analysis is to extend this approach and study large-scale Lurie direct control systems with norm-unbounded coefficients. For brevity, we will not involve the concept of the isolated subsystem but will directly give the Lyapunov function by employing the isolated subsystem and estimate the upper bound of its total derivative.

*Notation.* Throughout this paper, ||*x*|| denotes the Euclidean norm $||x||=\sqrt{x^{T}x}$, where $x={\left(\begin{array}{cccc} x_{1} & x_{2} & \cdots & x_{m} \end{array}\right)}^{T}$ is a column vector and *T* denotes the transposition. For vectors $x={\left(\begin{array}{cccc} x_{1} & x_{2} & \cdots & x_{m} \end{array}\right)}^{T}$, $y={\left(\begin{array}{cccc} y_{1} & y_{2} & \cdots & y_{m} \end{array}\right)}^{T}$, *x* ≤ *y*  (*x* < *y*) means that *x*_*i*_ ≤ *y*_*i*_  (*x*_*i*_ < *y*_*i*_)  (*i* = 1,2,…, *m*). ||*A*|| represents the Euclidean norm of a matrix *A* which is induced by the Euclidean vector norm ||*x*||; that is, ||*A*|| = max⁡_||*x*||=1_⁡||*Ax*||. *λ*(*A*) denotes an arbitrary eigenvalue of matrix *A*, and *A* > 0  (*A* < 0) means that *A* is positive definite (negative definite). The symbol *I* stands for the time interval (*τ*, +*∞*), where *τ* ∈ *R* or *τ* = −*∞*. $\overset{-}{\lim_{t\to +\infty }}f(t)$ represents the upper limit of the function *f*(*t*); that is, $\overset{-}{\lim_{t\to +\infty }}f(t)=\lim_{u\to +\infty }\sup_{t\geq u}\left(f(t)\right)$. When the limit of function exists, the upper limit must exist and be equal to its limit, so if we change the upper limit involved in this paper into the limit, the conclusions still hold. The reason why we employ an upper limit to describe the theorems and corollaries is that the upper limit makes the stability conditions less conservative.

## 2. Absolute Stability of Large-Scale Lurie Systems with Single Nonlinearity

Consider the following large-scale Lurie direct control system with time-varying coefficients and single nonlinearity:

(1) x˙i=∑j=1rAij(t)xj+bi(t)f(σ), (i=1,2,…,r),σ=∑j=1rcjT(t)xj,

where *x*_*i*_ ∈ *R* ^*n*_*i*_^  (*i* = 1,2,…, *r*) are the state, *b*_*i*_(*t*), *c*_*i*_(*t*) ∈ *R* ^*n*_*i*_^  (*i* = 1,2,…, *r*) are the vector function, *b*_*i*_(*t*) continuous and *c*_*i*_(*t*)  (*i* = 1,2,…, *r*) have derivative in time interval *I*, ∑_*i*=1_^*r*^ *n*_*i*_ = *n*, and *A*_*ij*_(*t*)  (*i*, *j* = 1,2,…, *r*) are *n*_*i*_ × *n*_*j*_ matrix functions and are continuous in *I*. The nonlinearity *f*(*σ*) is a continuous function and satisfies

(2) f(·)∈K[0,+∞) ={f(·) ∣ f(0)=0,0<σf(σ)<+∞,σ≠0}.

System (1) is said to be absolutely stable if its zero solution is globally asymptotically stable for any nonlinearity *f*(*σ*) ∈ *K*[0, +*∞*) [16, 17].

Basic assumptions about system (1) are proposed in the following.

(A1) We assume that there exist positive definite symmetrical constant matrices *P*_1_, *P*_2_,…, *P*_*r*_ such that

(3) λ[AiiT(t)Pi+PiAii(t)]≤−si(t)≤−si≤−s,           (i=1,2,…,r), ∀t>T,

where *T* ∈ *I*, *P*_*i*_ ∈ *R* ^*n*_*i*_×*n*_*i*_^, *s*_*i*_(*t*) > 0, *s*_*i*_ > 0  (*i* = 1,2,…, *r*) are known functions and constants, respectively, and *s* = min⁡(*s*_1_, *s*_2_,…, *s*_*r*_).

**Remark 1**

Compared with [23], condition A1 just guarantees the global asymptotic stability of ${\dot{x}}_{i}=A_{ii}(t)x_{i}\;(i=1,2,\ldots ,r)$, not all the linear part of the first equation of (1). So it is exceedingly weak.

(A2) We assume that

(4) $$\begin{array}{c} \overset{r}{\underset{i=1}{\sum }}c_{i}^{T}\left(t\right)b_{i}\left(t\right)\leq -g\left(t\right),\;\forall t>T, \end{array}$$

where *g*(*t*) > 0 is a known function.

**Remark 2**

In [23, 29], we know that *c*_*i*_^*T*^ *b*_*i*_ < 0 is the necessary condition for absolute stability in the case of Lurie direct control systems with constant coefficients.

(A3) We assume that

(5) 2||Pibi(t)||si(t)g(t)≤αi,  ||∑j=1rcjT(t)Aji(t)+c˙iT(t)||si(t)g(t)≤βi,               (i=1,2,…,r), ∀t>T,

where *α*_*i*_, *β*_*i*_  (*i* = 1,2,…, *r*) are constant.

(A4) We assume that

(6)        2||PiAij(t)||si(t)sj(t)≤γij,(i,j=1,2,…,r;i≠j),  ∀t>T,

where *γ*_*ij*_  (*i*, *j* = 1,2,…, *r*; *i* ≠ *j*) are constant.

As we know, the norm-unbounded and time-varying coefficients in system (1) are the main roadblocks in estimating the total time derivative of the Lyapunov function. But, by using the *s*_*i*_(*t*), *g*(*t*)  (*i* = 1,2,…, *r*) in conditions A1 and A2 and placing them in the denominator, the relative magnitude of the norm-unbounded coefficients can be restricted in conditions A3 and A4. That is, the “infinite” nature can be expressed by the “finite” form, which makes the study of the absolute stability of large-scale systems (1) feasible. Therefore, we have the following results.

**Theorem 3**

Under A1, A2, A3, and A4, the system described by (1) is absolutely stable if the matrix

(7) $$\begin{array}{c} G=\left(\begin{array}{ccccc} -1 & \gamma_{12} & \cdots & \gamma_{1r} & \alpha_{1}\\ \gamma_{21} & -1 & \cdots & \gamma_{2r} & \alpha_{2}\\ \cdots & \cdots & \cdots & \cdots & \cdots \\ \gamma_{r1} & \gamma_{r2} & \cdots & -1 & \alpha_{r}\\ \beta_{1} & \beta_{2} & \cdots & \beta_{r} & -1 \end{array}\right) \end{array}$$

is stable.

**Proof**

Choose a candidate Lyapunov function for system (1) as

(8) $$\begin{array}{c} V=\overset{r}{\underset{i=1}{\sum }}d_{i}x_{i}^{T}P_{i}x_{i}+d_{r+1}\overset{\sigma }{\underset{0}{\int }}f\left(\sigma \right)d\sigma , \end{array}$$

where *d*_1_,…, *d*_*r*_, *d*_*r*+1_ are positive numbers that will be determined later. From condition A1, we get that ∑_*i*=1_^*r*^ *d*_*i*_ *x*_*i*_^*T*^ *P*_*i*_ *x*_*i*_ is a positive definite quadratic form, and, by the properties of *f*(*σ*), we know that *V* in (8) is radially unbounded, is positive definite, and has an infinitesimal upper bound.

*Remark  3.* This is very different from the Lurie indirect control system. For the Lurie indirect control system, *σ* is an independent component of the state vector. Because of this, in order to guarantee that *V* in (8) is positive definite in *R* ^*n*+1^ and is radially unbounded, we need to also assume ∫_0_^±*∞*^ *f*(*σ*)*dσ* = +*∞* in [20–22].

Based on the decomposition theory of large-scale systems, and employing a similar modus operandi, we let

(9) $$\begin{array}{c} V_{i}=x_{i}^{T}P_{i}x_{i}\;\left(i=1,2,\ldots ,r\right);\\ V_{r+1}=\overset{\sigma }{\underset{0}{\int }}f\left(\sigma \right)d\sigma . \end{array}$$

Then, (8) can be written as

(10) $$\begin{array}{c} V=\overset{r+1}{\underset{i=1}{\sum }}d_{i}V_{i}=\left(d_{1},\ldots ,d_{r},d_{r+1}\right)\left(\begin{array}{c} V_{1}\\ \vdots \\ V_{r}\\ V_{r+1} \end{array}\right). \end{array}$$

Now, we calculate the time derivatives of *V*_1_, *V*_2_,…, *V*_*r*+1_ along the trajectories of (1), respectively, and then combine them to get the total time derivative of *V*. The time derivative of each *V*_*i*_  (*i* = 1,2,…, *r*) along the trajectories of (1) can be processed as

(11) V˙i|(1)=2xiTPix˙i=2xiTPi(∑j=1rAij(t)xj+bi(t)f(σ))=xiT(AiiT(t)Pi+PiAii(t))xi+2∑j=1j≠irxiTPiAij(t)xj+2xiTPibi(t)f(σ).

By taking the property of norm and using A1, A3, and A4, we obtain

(12) V˙i|(1)≤−si(t)||xi||2+2∑j=1j≠ir||PiAij(t)||||xi||||xj||+2||Pibi(t)||||xi|||f(σ)|=si(t)||xi||(−si(t)||xi||       +∑j=1j≠ir2||PiAij(t)||si(t)sj(t)sj(t)||xj||       +2||Pibi(t)||si(t)g(t)g(t)|f(σ)|)≤si(t)||xi||×(−si(t)||xi||+∑j=1j≠irγijsj(t)||xj||   +αig(t)|f(σ)|), ∀t>T.

On the other hand, the time derivative of *V*_*r*+1_ along the trajectories of (1) is given by

(13) V˙r+1|(1)=f(σ)σ˙|(1)=f(σ)(∑j=1rc˙jT(t)xj+∑j=1rcjT(t)x˙j)=f(σ) ×(∑j=1rc˙jT(t)xj    +∑j=1rcjT(t)(∑i=1rAji(t)xi+bj(t)f(σ)))=f(σ)(∑i=1r(∑j=1rcjT(t)Aji(t)+c˙iT(t))xi      +∑j=1rcjT(t)bj(t)f(σ)).

From A2 and A3, we have

(14) V˙r+1|(1)≤g(t)|f(σ)|∑i=1r||∑j=1rcjT(t)Aji(t)+c˙iT(t)||si(t)g(t)×si(t)||xi||−g(t)f2(σ)≤g(t)|f(σ)|×(∑i=1rβisi(t)||xi||−g(t)|f(σ)|),                ∀t>T.

Combining (12) with (14), we derive

(15) (V˙1⋮V˙rV˙r+1)(1)≤(s1(t)||x1||⋱sr(t)||xr||g(t)|f(σ)|) ×G(s1(t)||x1||⋮sr(t)||xr||g(t)|f(σ)|), ∀t>T.

Here, we define the following diagonal matrix:

(16) $$\begin{array}{c} D=\mathrm{diag}\left(d_{1},\ldots ,d_{r},d_{r+1}\right). \end{array}$$

Then, employing inequality (15), we get

(17) V˙|(1)=(d1,…,dr,dr+1)(V˙1⋮V˙rV˙r+1)(1)≤(d1,…,dr,dr+1)×(s1(t)||x1||⋱sr(t)||xr||g(t)|f(σ)|)×G(s1(t)||x1||⋮sr(t)||xr||g(t)|f(σ)|)=(s1(t)||x1||⋯sr(t)||xr||g(t)|f(σ)|)×GTD+DG2(s1(t)||x1||⋮sr(t)||xr||g(t)|f(σ)|), ∀t>T.

Since *G* is stable, this implies that the real part of each eigenvalue of −*G* is positive. From the equivalent propositions of the nonsingular *M*-matrix in [23, 30], we know that there exists a positive diagonal matrix *D* = diag⁡(*d*_1_,…, *d*_*r*_, *d*_*r*+1_)  (*d*_*i*_ > 0,  *i* = 1,…, *r*, *r* + 1) such that

(18) $$\begin{array}{c} \frac{{\left(-G\right)}^{T}D+D\left(-G\right)}{2} \end{array}$$

is positive definite; that is, ((*G* ^*T*^ *D* + *DG*)/2) is negative definite. Here, we choose the above *d*_1_,…, *d*_*r*_, *d*_*r*+1_ in (8) and let −*ω* be the biggest eigenvalue of ((*G* ^*T*^ *D* + *DG*)/2) (clearly −*ω* < 0). So, according to (17), we have

(19) V˙|(1)≤−ω(∑i=1rsi(t)||xi||2+g(t)f2(σ))≤−sω∑i=1r||xi||2,                         ∀t>T.

This implies that, as to all *f*(*σ*) ∈ *K*[0, +*∞*), $\dot{V}{|}_{\left(1\right)}$ is negative definite. Thus, according to the Lyapunov theorems, system (1) is absolutely stable. The proof is completed.

It should be noted that A3 and A4 can be weakened by establishing upper limits. Therefore, the following corollaries are obtained.

(A3′) Assume that

(20) $$\begin{array}{c} \underset{t\to +\infty }{\bar{\lim}}\frac{2||P_{i}b_{i}\left(t\right)||}{\sqrt{s_{i}\left(t\right)g\left(t\right)}}={\bar{\alpha }}_{i},\;\left(i=1,2,\ldots ,r\right),\\ \underset{t\to +\infty }{\bar{\lim}}\frac{||\sum_{j=1}^{r}c_{j}^{T}\left(t\right)A_{ji}\left(t\right)+{\dot{c}}_{i}^{T}\left(t\right)||}{\sqrt{s_{i}\left(t\right)g\left(t\right)}}={\bar{\beta }}_{i},\;\left(i=1,2,\ldots ,r\right), \end{array}$$

where ${\bar{\alpha }}_{i},{\bar{\beta }}_{i}$  (*i* = 1,2,…, *r*) are constant.

(A4′) Assume that

(21) $$\begin{array}{c} \underset{t\to +\infty }{\bar{\lim}}\frac{2||P_{i}A_{ij}\left(t\right)||}{\sqrt{s_{i}\left(t\right)s_{j}\left(t\right)}}={\bar{\gamma }}_{ij},\;\left(i,j=1,2,\ldots ,r;\;i\neq j\right), \end{array}$$

where ${\bar{\gamma }}_{ij}\;(i,j=1,2,\ldots ,r;i\neq j)$ are constant.

**Corollary 4**

Under A1, A2, A3′, and A4′, the system described by (1) is absolutely stable if the matrix

(22) $$\begin{array}{c} \bar{G}=\left(\begin{array}{ccccc} -1 & {\bar{\gamma }}_{12} & \cdots & {\bar{\gamma }}_{1r} & {\bar{\alpha }}_{1}\\ {\bar{\gamma }}_{21} & -1 & \cdots & {\bar{\gamma }}_{2r} & {\bar{\alpha }}_{2}\\ \cdots & \cdots & \cdots & \cdots & \cdots \\ {\bar{\gamma }}_{r1} & {\bar{\gamma }}_{r2} & \cdots & -1 & {\bar{\alpha }}_{r}\\ {\bar{\beta }}_{1} & {\bar{\beta }}_{2} & \cdots & {\bar{\beta }}_{r} & -1 \end{array}\right) \end{array}$$

is stable.

**Proof**

Let

(23) $$\begin{array}{c} G=\left(\begin{array}{ccccc} -1 & {\bar{\gamma }}_{12}+\varepsilon & \cdots & {\bar{\gamma }}_{1r}+\varepsilon & {\bar{\alpha }}_{1}+\varepsilon \\ {\bar{\gamma }}_{21}+\varepsilon & -1 & \cdots & {\bar{\gamma }}_{2r}+\varepsilon & {\bar{\alpha }}_{2}+\varepsilon \\ \cdots & \cdots & \cdots & \cdots & \cdots \\ {\bar{\gamma }}_{r1}+\varepsilon & {\bar{\gamma }}_{r2}+\varepsilon & \cdots & -1 & {\bar{\alpha }}_{r}+\varepsilon \\ {\bar{\beta }}_{1}+\varepsilon & {\bar{\beta }}_{2}+\varepsilon & \cdots & {\bar{\beta }}_{r}+\varepsilon & -1 \end{array}\right). \end{array}$$

Namely, *G* is a matrix induced by $\bar{G}$ whose every off-diagonal entry is increased by *ε*.

According to the properties of the matrix, if $\bar{G}$ is stable, then there exists a sufficiently small scalar *ε* > 0 such that *G* is also stable. We choose an *ε* > 0 that can allow for the stability of *G*.

From the assumptions here and the definition of upper limit, as to the above *ε*, there exists a number *T*(≥*τ*), which satisfies that if *t* > *T*, then

(24) 2||Pibi(t)||si(t)g(t)≤sup⁡t>T(2||Pibi(t)||si(t)g(t))≤α¯i+ε,                (i=1,2,…,r)||∑j=1rcjT(t)Aji(t)+c˙iT(t)||si(t)g(t)≤sup⁡t>T(||∑j=1rcjT(t)Aji(t)+c˙iT(t)||si(t)g(t))≤β¯i+ε,                  (i=1,2,…,r),2||PiAij(t)||si(t)sj(t)≤sup⁡t>T(2||PiAij(t)||si(t)sj(t))≤γ¯ij+ε,             (i,j=1,2,…,r;i≠j).

Thus, according to Theorem 3, system (1) is absolutely stable.

If ${\bar{\gamma }}_{ij}=0\;(i,j=1,2,\ldots ,r;i\neq j)$, then the following corollary is proposed.

(A4′′) We assume that

(25) $$\begin{array}{c} \underset{t\to +\infty }{\bar{\lim}}\frac{2||P_{i}A_{ij}\left(t\right)||}{\sqrt{s_{i}\left(t\right)s_{j}\left(t\right)}}=0,\;\left(i,j=1,2,\ldots ,r;i\neq j\right). \end{array}$$

Namely, ${\bar{\gamma }}_{ij}=0\;(i,j=1,2,\ldots ,r;i\neq j)$ in condition A4′.

**Corollary 5**

Under A1, A2, A3′, and A4′′, the system described by (1) is absolutely stable if the inequality $\sum_{i=1}^{r}{\bar{\alpha }}_{i}{\bar{\beta }}_{i}<1$ holds.

**Proof**

According to A4′′, the eigenpolynomial of the matrix $\bar{G}$ can be obtained as

(26) |λI−G¯|=|λ+10⋯0−α¯10λ+1⋯0−α¯2⋯⋯⋯⋯⋯00⋯λ+1−α¯r−β¯1−β¯2⋯−β¯rλ+1|=(λ2+2λ+(1−∑i=1rα¯iβ¯i))(λ+1)r−1,

where *λ* = −1 is an eigenvalue with multiplicity (*r* − 1) and the other two eigenvalues satisfy

(27) $$\begin{array}{c} \lambda^{2}+2\lambda +\left(1-\overset{r}{\underset{i=1}{\sum }}{\bar{\alpha }}_{i}{\bar{\beta }}_{i}\right)=0. \end{array}$$

It is easy to see that all roots of the above equation have a real part if and only if $\sum_{i=1}^{r}{\bar{\alpha }}_{i}{\bar{\beta }}_{i}<1$. So $\bar{G}$ is stable if $\sum_{i=1}^{r}{\bar{\alpha }}_{i}{\bar{\beta }}_{i}<1$. This implies that system (1) is absolutely stable by Corollary 4.

Consider a more specific case; we have the following corollary.

**Corollary 6**

Under A1, A2, A3′, and A4′, the system described by (1) is absolutely stable if

(28) $$\begin{array}{c} {\bar{\alpha }}_{i}={\bar{\gamma }}_{ij}=0,\;\left(i,j=1,2,\ldots ,r;i\neq j\right) \end{array}$$

or

(29) $$\begin{array}{c} {\bar{\beta }}_{i}={\bar{\gamma }}_{ij}=0,\;\left(i,j=1,2,\ldots ,r;i\neq j\right) \end{array}$$

holds.

**Proof**

From $\sum_{i=1}^{r}{\bar{\alpha }}_{i}{\bar{\beta }}_{i}=0<1$, we know that system (1) is absolutely stable by Corollary 5.

**Corollary 7**

Under A1, A2, A3′, and A4′, the system described by (1) is absolutely stable if the following inequalities hold:

(30) $$\begin{array}{c} \overset{r}{\underset{i=1}{\sum }}\left({\bar{\alpha }}_{i}+{\bar{\beta }}_{i}\right)<2,\\ {\bar{\alpha }}_{j}+{\bar{\beta }}_{j}+\overset{r}{\underset{\begin{array}{c} i=1\\ i\neq j \end{array}}{\sum }}\left({\bar{\gamma }}_{ji}+{\bar{\gamma }}_{ij}\right)<2,\;\left(j=1,2,\ldots ,r\right). \end{array}$$

**Proof**

Choosing the undetermined coefficients of the Lyapunov function in Theorem 3 as *d*_1_ = ⋯ = *d*_*r*_ = *d*_*r*+1_ = 1 and combining them with Corollary 4, we just need to prove that

(31) $$\begin{array}{c} \bar{G}+{\bar{G}}^{T}=\left(\begin{array}{ccccc} -2 & {\bar{\gamma }}_{12}+{\bar{\gamma }}_{21} & \cdots & {\bar{\gamma }}_{1r}+{\bar{\gamma }}_{r1} & {\bar{\alpha }}_{1}+{\bar{\beta }}_{1}\\ {\bar{\gamma }}_{12}+{\bar{\gamma }}_{21} & -2 & \cdots & {\bar{\gamma }}_{2r}+{\bar{\gamma }}_{r2} & {\bar{\alpha }}_{2}+{\bar{\beta }}_{2}\\ \cdots & \cdots & \cdots & \cdots & \cdots \\ {\bar{\gamma }}_{1r}+{\bar{\gamma }}_{r1} & {\bar{\gamma }}_{2r}+{\bar{\gamma }}_{r2} & \cdots & -2 & {\bar{\alpha }}_{r}+{\bar{\beta }}_{r}\\ {\bar{\alpha }}_{1}+{\bar{\beta }}_{1} & {\bar{\alpha }}_{2}+{\bar{\beta }}_{2} & \cdots & {\bar{\alpha }}_{r}+{\bar{\beta }}_{r} & -2 \end{array}\right) \end{array}$$

is negative definite. Note that each eigenvalue of $\bar{G}+{\bar{G}}^{T}$ is a real number (since $\bar{G}+{\bar{G}}^{T}$ is a real symmetric matrix) and every diagonal entry is equal to 2, so, from the Gershgorin circle theorem in [31], we get

(32) $$\begin{array}{c} \left|\lambda +2\right|\leq \overset{r}{\underset{i=1}{\sum }}\left({\bar{\alpha }}_{i}+{\bar{\beta }}_{i}\right),\\ \left|\lambda +2\right|\leq {\bar{\alpha }}_{j}+{\bar{\beta }}_{j}+\overset{r}{\underset{\begin{array}{c} i=1\\ i\neq j \end{array}}{\sum }}\left({\bar{\gamma }}_{ji}+{\bar{\gamma }}_{ij}\right),\;\left(j=1,2,\ldots ,r\right). \end{array}$$

Namely,

(33) $$\begin{array}{c} \lambda \leq -2+\overset{r}{\underset{i=1}{\sum }}\left({\bar{\alpha }}_{i}+{\bar{\beta }}_{i}\right),\\ \lambda \leq -2+{\bar{\alpha }}_{j}+{\bar{\beta }}_{j}+\overset{r}{\underset{\begin{array}{c} i=1\\ i\neq j \end{array}}{\sum }}\left({\bar{\gamma }}_{ji}+{\bar{\gamma }}_{ij}\right),\;\left(j=1,2,\ldots ,r\right). \end{array}$$

If inequalities (30) hold, then each eigenvalue *λ* of $\bar{G}+{\bar{G}}^{T}$ satisfies *λ* < 0, and this implies that $\bar{G}+{\bar{G}}^{T}$ is negative definite. The proof is completed.

Moreover, the conditions of Corollary 7 can be weakened as follows.

**Corollary 8**

Under A1, A2, A3′, and A4′, the system described by (1) is absolutely stable if the following inequalities

(34) $$\begin{array}{c} \overset{r}{\underset{i=1}{\sum }}{\bar{\alpha }}_{i}<1,\\ {\bar{\beta }}_{j}+\overset{r}{\underset{\begin{array}{c} i=1\\ i\neq j \end{array}}{\sum }}{\bar{\gamma }}_{ij}<1,\;\left(j=1,2,\ldots ,r\right) \end{array}$$

or

(35) $$\begin{array}{c} \overset{r}{\underset{i=1}{\sum }}{\bar{\beta }}_{i}<1,\\ {\bar{\alpha }}_{j}+\overset{r}{\underset{\begin{array}{c} i=1\\ i\neq j \end{array}}{\sum }}{\bar{\gamma }}_{ji}<1,\;\left(j=1,2,\ldots ,r\right) \end{array}$$

hold.

**Proof**

From the Gershgorin circle theorem, we know that each eigenvalue *λ* of $\bar{G}$ satisfies

(36) $$\begin{array}{c} \left|\lambda +1\right|\leq \overset{r}{\underset{i=1}{\sum }}{\bar{\alpha }}_{i},\\ \left|\lambda +1\right|\leq {\bar{\beta }}_{j}+\overset{r}{\underset{\begin{array}{c} i=1\\ i\neq j \end{array}}{\sum }}{\bar{\gamma }}_{ij},\;\left(j=1,2,\ldots ,r\right). \end{array}$$

Then, we have

(37) |Re(λ)+1|≤|λ+1|≤∑i=1rα¯i,|Re(λ)+1|≤|λ+1|≤β¯j+∑i=1i≠jrγ¯ij, (j=1,2,…,r).

Namely,

(38) Re(λ)≤−1+∑i=1rα¯i,Re(λ)≤−1+β¯j+∑i=1i≠jrγ¯ij, (j=1,2,…,r).

If inequalities (34) hold, then *Re*(*λ*) < 0, and this implies that $\bar{G}$ is stable. Similarly, if inequalities (35) hold, we can prove that ${\bar{G}}^{T}$ is stable; that is, $\bar{G}$ is stable. With the conditions of Corollary 4 being satisfied, we conclude that system (1) is absolutely stable.

**Remark 9**

If inequalities (34) and (35) hold together, inequalities (30) hold. Therefore, Corollary 8 is less conservative.

## 3. Absolute Stability of Large-Scale Lurie Systems with Multiple Nonlinearities

Consider the following large-scale Lurie direct control systems with time-varying coefficients and multiple nonlinearities:

(39) $$\begin{array}{c} {\dot{x}}_{i}=\overset{r}{\underset{j=1}{\sum }}A_{ij}\left(t\right)x_{j}+\overset{m}{\underset{k=1}{\sum }}b_{ik}\left(t\right)f_{k}\left(\sigma_{k}\right),\\ \sigma_{k}=\overset{r}{\underset{j=1}{\sum }}c_{kj}^{T}\left(t\right)x_{j},\\ \left(i,j=1,2,\ldots ,r;k=1,2,\ldots ,m\right), \end{array}$$

where *x*_*i*_ ∈ *R* ^*n*_*i*_^  (*i* = 1,2,…, *r*) are the state, *b*_*ik*_(*t*) ∈ *R* ^*n*_*i*_^  (*i* = 1,2,…, *r*; *k* = 1,2,…, *m*), *c*_*kj*_(*t*) ∈ *R* ^*n*_*i*_^  (*k* = 1,2,…, *m*; *j* = 1,2,…, *r*) are vector functions, *b*_*ik*_(*t*) continuous and *c*_*kj*_(*t*) have derivatives in time interval *I*, ∑_*i*=1_^*r*^ *n*_*i*_ = *n*, and  *A*_*ij*_(*t*)  (*i*, *j* = 1,2,…, *r*) are *n*_*i*_ × *n*_*j*_ matrix functions and are continuous in *I*. The nonlinearities *f*_*k*_(·)  (*k* = 1,2,…, *m*) are continuous functions, and they satisfy

(40) fk(·)∈K[0,+∞) ={fk(·) ∣ fk(0)=0,0<σkfk(σk)<+∞,σk≠0}.

System (39) is said to be absolutely stable if its zero solution is globally asymptotically stable for any nonlinearity *f*_*k*_(*σ*_*k*_) ∈ *K*[0, +*∞*) [16, 17].

Basic assumptions about system (39) are proposed.

(A5) We assume that there exist positive definite symmetrical constant matrices *P*_1_, *P*_2_,…, *P*_*r*_ such that

(41) λ[AiiT(t)Pi+PiAii(t)]≤−si(t)≤−si≤−s,           (i=1,2,…,r), ∀t>T,

where *T* ∈ *I*, *s*_*i*_(*t*) > 0, *s*_*i*_ > 0  (*i* = 1,2,…, *r*) are known functions and constants, respectively, and *s* = min⁡(*s*_1_, *s*_2_,…, *s*_*r*_).

(A6) We assume that

(42) ∑j=1rckjT(t)bjk(t)≤−gk(t),(k=1,2,…,m), ∀t>T,

where *g*_*k*_(*t*) > 0  (*k* = 1,2,…, *m*) are known functions.

(A7) We assume that

(43) 2||Pibik(t)||si(t)gk(t)≤αik,||∑j=1rckjT(t)Aji(t)+c˙kiT(t)||si(t)gk(t)≤βki,       (i=1,2,…,r;k=1,2,…,m), ∀t>T,

where *α*_*ik*_, *β*_*ki*_  (*i* = 1,2,…, *r*; *k* = 1,2,…, *m*) are constants.

(A8) We assume that

(44)        2||PiAij(t)||si(t)sj(t)≤γij,(i,j=1,2,…,r;i≠j), ∀t>T,

where *γ*_*ij*_  (*i*, *j* = 1,2,…, *r*; *i* ≠ *j*) are constants.

(A9) We assume that

(45) $$\begin{array}{c} \frac{\left|\sum_{j=1}^{r}c_{kj}^{T}\left(t\right)b_{jl}\left(t\right)\right|}{\sqrt{g_{k}\left(t\right)g_{l}\left(t\right)}}\leq \mu_{kl},\;\left(k,l=1,2,\ldots ,m;k\neq l\right),\;\;\forall t>T, \end{array}$$

where *μ*_*kl*_  (*k*, *l* = 1,2,…, *m*; *k* ≠ *l*) are constants.

In addition, we define the following matrices:

(46) $$\begin{array}{c} G=\left(\begin{array}{cccc} -1 & \gamma_{12} & \cdots & \gamma_{1r}\\ \gamma_{21} & -1 & \cdots & \gamma_{2r}\\ \cdots & \cdots & \cdots & \cdots \\ \gamma_{r1} & \gamma_{r2} & \cdots & -1 \end{array}\right),\\ F=\left(\begin{array}{cccc} -1 & \mu_{12} & \cdots & \mu_{1m}\\ \mu_{21} & -1 & \cdots & \mu_{2m}\\ \cdots & \cdots & \cdots & \cdots \\ \mu_{m1} & \mu_{m2} & \cdots & -1 \end{array}\right),\\ R=\left(\begin{array}{cccc} \alpha_{11} & \alpha_{12} & \cdots & \alpha_{1m}\\ \alpha_{21} & \alpha_{22} & \cdots & \alpha_{2m}\\ \cdots & \cdots & \cdots & \cdots \\ \alpha_{r1} & \alpha_{r2} & \cdots & \alpha_{rm} \end{array}\right),\\ L=\left(\begin{array}{cccc} \beta_{11} & \beta_{12} & \cdots & \beta_{1r}\\ \beta_{21} & \beta_{22} & \cdots & \beta_{2r}\\ \cdots & \cdots & \cdots & \cdots \\ \beta_{m1} & \beta_{m2} & \cdots & \beta_{mr} \end{array}\right). \end{array}$$

**Theorem 10**

Under A5, A6, A7, A8, and A9, the system described by (39) is absolutely stable if the matrix

(47) $$\begin{array}{c} Q=\left(\begin{array}{cc} G & R\\ L & F \end{array}\right) \end{array}$$

is stable.

**Proof**

Choose a candidate Lyapunov function for system (39) as

(48) $$\begin{array}{c} V=\overset{r}{\underset{i=1}{\sum }}d_{i}x_{i}^{T}P_{i}x_{i}+d_{r+1}\overset{\sigma }{\underset{0}{\int }}f\left(\sigma \right)d\sigma , \end{array}$$

where *d*_1_,…, *d*_*r*_, *d*_*r*+1_,…, *d*_*r*+*m*_ are positive numbers that will be chosen later. From the properties of *f*_*k*_(*σ*_*k*_) and condition A5, we know that *V* in (48) is radially unbounded, is positive definite, and has infinitesimal upper bound.

Let

(49) $$\begin{array}{c} V_{i}=x_{i}^{T}P_{i}x_{i}\;\left(i=1,2,\ldots r\right),\\ V_{r+k}=\overset{\sigma_{k}}{\underset{0}{\int }}f_{k}\left(\sigma \right)d\sigma \;\left(k=1,2,\ldots m\right). \end{array}$$

Then, (48) can be written as

(50) $$\begin{array}{c} V=\overset{r+m}{\underset{i=1}{\sum }}d_{i}V_{i}=\left(\begin{array}{cccccc} d_{1} & \cdots & d_{r} & d_{r+1} & \cdots & d_{r+m} \end{array}\right)\left(\begin{array}{c} V_{1}\\ \vdots \\ V_{r}\\ V_{r+1}\\ \vdots \\ V_{r+m} \end{array}\right). \end{array}$$

First, we calculate the time derivative of each *V*_*i*_  (*i* = 1,2,…, *r*) along the trajectories of system (39). Consider

(51) V˙i|(39)=2xiTPix˙i=2xiTPi(∑j=1rAij(t)xj+∑k=1mbik(t)fk(σk))=xiT(AiiT(t)Pi+PiAii(t))xi+2∑j=1j≠irxiTPiAij(t)xj+2∑k=1mxiTPibik(t)fk(σk).

From A5, A7, and A8, we have

(52) V˙i|(39)≤−si(t)||xi||2+2∑j=1j≠ir||PiAij(t)||||xi||||xj||+2∑k=1m||Pibik(t)||||xi|||fk(σk)|=si(t)||xi||×(−si(t)||xi||+∑j=1j≠ir2||PiAij(t)||si(t)sj(t)sj(t)||xj||   +∑k=1m2||Pibik(t)||si(t)gk(t)gk(t)|fk(σk)|)≤si(t)||xi||×(−si(t)||xi||+∑j=1j≠irγijsj(t)||xj||   +∑k=1mαikgk(t)|fk(σk)|), ∀t>T.

Second, the time derivative of each *V*_*r*+*k*_  (*k* = 1,2,…, *m*) along the trajectories of (39) is given as

(53) V˙r+k|(39)=fk(σk)σ˙k|(39)=fk(σk)(∑j=1rc˙kjT(t)xj+∑j=1rckjT(t)x˙j)=fk(σk)(∑j=1rc˙kjT(t)xj       +∑j=1rckjT(t)(∑i=1rAji(t)xi+∑l=1mbjl(t)fl(σl)))=fk(σk)(∑i=1r(∑j=1rckjT(t)Aji(t)+c˙kiT(t))xi       +∑l=1m∑j=1rckjT(t)bjl(t)fl(σl)).

From A6, A7, and A9, we get

(54) V˙r+k|(39)≤gk(t)|fk(σk)| ×∑i=1r||∑j=1rckjT(t)Aji(t)+c˙kiT(t)||si(t)gk(t)si(t)||xi|| −gk(t)fk2(σk) +gk(t)|fk(σk)|∑l=1l≠km|∑j=1rckjT(t)bjl(t)|gk(t)gl(t)gl(t)|fl(σl)|≤gk(t)|fk(σk)| ×(∑i=1rβkisi(t)||xi||−gk(t)|fk(σk)|    +∑l=1l≠kmμklgl(t)|fl(σl)|), ∀t>T.

Then, we combine (52) with (54) to obtain

(55) $$\begin{array}{c} {\left(\begin{array}{c} {\dot{V}}_{1}\\ \vdots \\ {\dot{V}}_{r}\\ {\dot{V}}_{r+1}\\ \vdots \\ {\dot{V}}_{r+m} \end{array}\right)}_{\left(39\right)}<\text{WQU},\;\forall t>T, \end{array}$$

where

(56) $$\begin{array}{c} W=\left(\begin{array}{cccccc} \sqrt{s_{1}\left(t\right)}||x_{1}|| & & & & & \\ & \ddots & & & & \\ & & \sqrt{s_{r}\left(t\right)}||x_{r}|| & & & \\ & & & \sqrt{g_{1}\left(t\right)}\left|f_{1}\left(\sigma_{1}\right)\right| & & \\ & & & & \ddots & \\ & & & & & \sqrt{g_{m}\left(t\right)}\left|f_{m}\left(\sigma_{m}\right)\right| \end{array}\right),\\ U={\left(\begin{array}{cccccc} \sqrt{s_{1}(t)}||x_{1}|| & \cdots & \sqrt{s_{r}(t)}||x_{r}|| & \sqrt{g_{1}(t)}\left|f_{1}(\sigma_{1})\right| & \cdots & \sqrt{g_{m}(t)}\left|f_{m}(\sigma_{m})\right| \end{array}\right)}^{T}. \end{array}$$

Let *D* = diag⁡(*d*_1_,…, *d*_*r*_, *d*_*r*+1_,…, *d*_*r*+*m*_), and use (55) to obtain

(57) V˙|(39)=(d1⋯drdr+1⋯dr+m)(V˙1⋮V˙rV˙r+1⋮V˙r+m)(39)≤(d1,d2,…,dr+m)WQU=UTQTD+DQ2U, ∀t>T.

Similarly as with Theorem 3, we can prove that there exists a positive diagonal matrix *D* = diag⁡(*d*_1_, *d*_2_,…, *d*_*r*+*m*_)  (*d*_*i*_ > 0, *i* = 1,2,…, *r* + *m*) such that ((*Q* ^*T*^ *D* + *DQ*)/2) is negative definite. Choose the above *d*_1_, *d*_2_,…, *d*_*r*+*m*_ in (48), and let −*ω* be the biggest eigenvalue of (1/2)(*Q* ^*T*^ *D* + *DQ*) (clearly −*ω* < 0). Hence, according to (57), we have

(58) V˙|(39)≤−ω||U||2=−ω(∑i=1rsi(t)||xi||2+∑k=1mgk(t)fk2(σk))≤−sω∑i=1r||xi||2, ∀t>T.

This implies that, as to all *f*_*k*_(*σ*_*k*_) ∈ *K*[0, +*∞*), $\dot{V}{|}_{\left(39\right)}$ is negative definite. So, system (39) is absolutely stable by the Lyapunov theorems. The proof is completed.

At the same time, we can get the following corollaries. The proof for corollaries is similar to that in large-scale Lurie systems with single nonlinearity and thus is omitted.

(A7′) We assume that

(59)    lim⁡¯x→+∞2||Pibik(t)||si(t)gk(t)=α¯ik,(i=1,2,…,r;k=1,2,…,m),lim⁡¯x→+∞||∑j=1rckjT(t)Aji(t)+c˙kiT(t)||si(t)gk(t)=β¯ki,      (i=1,2,…,r;k=1,2,…,m),

where$\;{\bar{\alpha }}_{ik},{\bar{\beta }}_{ki}\;(i=1,2,\ldots ,r;k=1,2,\ldots ,m)\;$are constant.

(A8′) We assume that

(60) $$\begin{array}{c} \underset{x\to +\infty }{\bar{\lim}}\frac{2||P_{i}A_{ij}\left(t\right)||}{\sqrt{s_{i}\left(t\right)s_{j}\left(t\right)}}={\bar{\gamma }}_{ij},\;\left(i,j=1,2,\ldots ,r;i\neq j\right), \end{array}$$

where$\;{\bar{\gamma }}_{ij}\;(i,j=1,2,\ldots ,r;i\neq j)\;$are constant.

(A9′) We assume that

(61) $$\begin{array}{c} \underset{t\to +\infty }{\lim}\frac{\left|\sum_{j=1}^{r}c_{kj}^{T}\left(t\right)b_{jl}\left(t\right)\right|}{\sqrt{g_{k}\left(t\right)g_{l}\left(t\right)}}={\bar{\mu }}_{kl},\;\left(k,l=1,2,\ldots ,m;k\neq l\right), \end{array}$$

where $\;{\bar{\mu }}_{kl}\;(k,l=1,2,\ldots ,m;k\neq l)\;$ are constant.

**Corollary 11**

Under A5, A6, A7′, A8′, and A9′, the system described by (39) is absolutely stable if the matrix

(62) $$\begin{array}{c} \bar{Q}=\left(\begin{array}{cc} \bar{G} & \bar{R}\\ \bar{L} & \bar{F} \end{array}\right) \end{array}$$

is stable, where

(63) $$\begin{array}{c} \bar{G}=\left(\begin{array}{cccc} -1 & {\bar{\gamma }}_{12} & \cdots & {\bar{\gamma }}_{1r}\\ {\bar{\gamma }}_{21} & -1 & \cdots & {\bar{\gamma }}_{2r}\\ \cdots & \cdots & \cdots & \cdots \\ {\bar{\gamma }}_{r1} & {\bar{\gamma }}_{r2} & \cdots & -1 \end{array}\right),\\ \bar{F}=\left(\begin{array}{cccc} -1 & {\bar{\mu }}_{12} & \cdots & {\bar{\mu }}_{1m}\\ {\bar{\mu }}_{21} & -1 & \cdots & {\bar{\mu }}_{2m}\\ \cdots & \cdots & \cdots & \cdots \\ {\bar{\mu }}_{m1} & {\bar{\mu }}_{m2} & \cdots & -1 \end{array}\right),\\ \bar{R}=\left(\begin{array}{cccc} {\bar{\alpha }}_{11} & {\bar{\alpha }}_{12} & \cdots & {\bar{\alpha }}_{1m}\\ {\bar{\alpha }}_{21} & {\bar{\alpha }}_{22} & \cdots & {\bar{\alpha }}_{2m}\\ \cdots & \cdots & \cdots & \cdots \\ {\bar{\alpha }}_{r1} & {\bar{\alpha }}_{r2} & \cdots & {\bar{\alpha }}_{rm} \end{array}\right),\\ \bar{L}=\left(\begin{array}{cccc} {\bar{\beta }}_{11} & {\bar{\beta }}_{12} & \cdots & {\bar{\beta }}_{1r}\\ {\bar{\beta }}_{21} & {\bar{\beta }}_{22} & \cdots & {\bar{\beta }}_{2r}\\ \cdots & \cdots & \cdots & \cdots \\ {\bar{\beta }}_{m1} & {\bar{\beta }}_{m2} & \cdots & {\bar{\beta }}_{mr} \end{array}\right). \end{array}$$

**Corollary 12**

Under A5, A6, A8′, and A9′ and with matrices $\;\bar{G}$, $\bar{F}\;$ being stable, the system described by (1) is absolutely stable if

(64) $$\begin{array}{c} \underset{t\to +\infty }{\lim}\frac{2||P_{i}b_{ik}\left(t\right)||}{\sqrt{s_{i}\left(t\right)g_{k}\left(t\right)}}=0,\\ \frac{||\sum_{j=1}^{r}c_{kj}^{T}\left(t\right)A_{ji}\left(t\right)+{\dot{c}}_{ki}^{T}\left(t\right)||}{\sqrt{s_{i}\left(t\right)g_{k}\left(t\right)}}\leq M_{ki},\\ \left(i=1,2,\ldots ,r;k=1,2,\ldots ,m\right) \end{array}$$

or

(65) $$\begin{array}{c} \frac{2||P_{i}b_{ik}\left(t\right)||}{\sqrt{s_{i}\left(t\right)g_{k}\left(t\right)}}\leq K_{ik},\\ \underset{t\to +\infty }{\lim}\frac{||\sum_{j=1}^{r}c_{kj}^{T}\left(t\right)A_{ji}\left(t\right)+{\dot{c}}_{ki}^{T}\left(t\right)||}{\sqrt{s_{i}\left(t\right)g_{k}\left(t\right)}}=0,\\ \left(i=1,2,\ldots ,r;k=1,2,\ldots ,m\right) \end{array}$$

holds, where *M*_*ki*_, *K*_*ik*_  (*i* = 1,2,…, *r*; *k* = 1,2,…, *m*) are constant.

**Corollary 13**

Under A5, A6, A7′, A8′, and A9′, the system described by (39) is absolutely stable if the following inequalities

(66) $$\begin{array}{c} \overset{r}{\underset{\begin{array}{c} j=1\\ i\neq j \end{array}}{\sum }}{\bar{\gamma }}_{ij}+\overset{m}{\underset{j=1}{\sum }}{\bar{\alpha }}_{ij}<1,\;\left(i=1,2,\ldots ,r\right),\\ \overset{m}{\underset{\begin{array}{c} j=1\\ k\neq j \end{array}}{\sum }}{\bar{\mu }}_{kj}+\overset{r}{\underset{j=1}{\sum }}{\bar{\beta }}_{kj}<1,\;\left(k=1,2,\ldots ,m\right), \end{array}$$

or

(67) $$\begin{array}{c} \overset{r}{\underset{\begin{array}{c} i=1\\ i\neq j \end{array}}{\sum }}{\bar{\gamma }}_{ij}+\overset{m}{\underset{i=1}{\sum }}{\bar{\beta }}_{ij}<1,\;\left(j=1,2,\ldots ,r\right),\\ \overset{m}{\underset{\begin{array}{c} i=1\\ i\neq l \end{array}}{\sum }}{\bar{\mu }}_{il}+\overset{r}{\underset{i=1}{\sum }}{\bar{\alpha }}_{il}<1,\;\left(l=1,2,\ldots ,m\right), \end{array}$$

hold.

## 4. Numerical Examples

In this section, two simple numerical examples are introduced to demonstrate the effectiveness of our criteria.

**Example**

Consider system (1) with

(68) $$\begin{array}{c} A_{11}\left(t\right)=\left(\begin{array}{cc} -2t & 1\\ t & -3t \end{array}\right),\;\;A_{12}\left(t\right)=\left(\begin{array}{c} 1\\ t^{2} \end{array}\right),\\ A_{21}\left(t\right)=\left(\begin{array}{cc} t^{3} & -2t \end{array}\right),\;\;A_{22}\left(t\right)=-5t^{5},\\ b_{1}=\left(\begin{array}{c} -e^{t^{2}}\\ 2t \end{array}\right),\;\;b_{2}=-6t^{3};\\ c_{1}=\left(\begin{array}{cc} -e^{t^{2}} & 2t \end{array}\right),\;\;c_{2}=-6t^{3}. \end{array}$$

Note that the norms of coefficient matrices for the above system are unbounded; we choose

(69) $$\begin{array}{c} T=1,\;\;P_{1}=\left(\begin{array}{cc} 1 & 0\\ 0 & 1 \end{array}\right),\;\;P_{2}=1. \end{array}$$

Therefore,

(70) $$\begin{array}{c} s_{1}\left(t\right)=2t,\;s_{1}=2;\;\;s_{2}\left(t\right)=10t^{5},\;\;s_{2}=10;\\ g\left(t\right)=e^{2t^{2}}-2t^{3}. \end{array}$$

This means that assumptions A1 and A2 are satisfied. From

(71) $$\begin{array}{c} \underset{t\to +\infty }{\bar{\lim}}\frac{2||P_{1}b_{1}\left(t\right)||}{\sqrt{s_{1}\left(t\right)g\left(t\right)}}={\bar{\alpha }}_{1}=0,\\ \underset{t\to +\infty }{\bar{\lim}}\frac{2||P_{2}b_{2}\left(t\right)||}{\sqrt{s_{2}\left(t\right)g\left(t\right)}}={\bar{\alpha }}_{2}=0,\\ \underset{t\to +\infty }{\bar{\lim}}\frac{||\sum_{j=1}^{2}c_{j}^{T}\left(t\right)A_{j1}\left(t\right)+{\dot{c}}_{1}^{T}\left(t\right)||}{\sqrt{s_{1}\left(t\right)g\left(t\right)}}={\bar{\beta }}_{1}=0,\\ \underset{t\to +\infty }{\bar{\lim}}\frac{||\sum_{j=1}^{2}c_{j}^{T}\left(t\right)A_{j2}\left(t\right)+{\dot{c}}_{2}^{T}\left(t\right)||}{\sqrt{s_{2}\left(t\right)g\left(t\right)}}={\bar{\beta }}_{2}=0, \end{array}$$

we know that$\;{\bar{\alpha }}_{i}=0,\;\;{\bar{\beta }}_{i}=0\;(i=1,2)\;$in assumption A3′. Since

(72) $$\begin{array}{c} \underset{t\to +\infty }{\bar{\lim}}\frac{2||P_{1}A_{12}\left(t\right)||}{\sqrt{s_{1}\left(t\right)s_{2}\left(t\right)}}={\bar{\gamma }}_{12}=0,\\ \underset{t\to +\infty }{\bar{\lim}}\frac{2||P_{2}A_{21}\left(t\right)||}{\sqrt{s_{2}\left(t\right)s_{1}\left(t\right)}}={\bar{\gamma }}_{21}=\frac{1}{\sqrt{5}}, \end{array}$$

we know that assumption A4′ is satisfied and

(73) $$\begin{array}{c} \bar{G}=\left(\begin{array}{ccc} -1 & {\bar{\gamma }}_{12} & {\bar{\alpha }}_{1}\\ {\bar{\gamma }}_{21} & -1 & {\bar{\alpha }}_{2}\\ {\bar{\beta }}_{1} & {\bar{\beta }}_{2} & -1 \end{array}\right)=\left(\begin{array}{ccc} -1 & 0 & 0\\ \frac{1}{\sqrt{5}} & -1 & 0\\ 0 & 0 & -1 \end{array}\right). \end{array}$$

It is easy to verify that $\;\bar{G}\;$ is stable. In summary, according to Corollary 4 (or Corollary 8), the system is absolutely stable.

Let *f*(*σ*) = *σ* ^3^ and $\;x_{1}=\left(\begin{array}{c} y_{1}\\ y_{2} \end{array}\right),x_{2}=y_{3}$; then system (1) can be written as

(74) $$\begin{array}{c} {\dot{y}}_{1}=-2ty_{1}+y_{2}+y_{3}-e^{t^{2}}{\left(e^{t^{2}}y_{1}+t^{2}y_{2}+ty_{3}\right)}^{3},\\ {\dot{y}}_{2}=ty_{1}-3ty_{2}+t^{2}y_{3}+2t{\left(e^{t^{2}}y_{1}+t^{2}y_{2}+ty_{3}\right)}^{3},\\ {\dot{y}}_{3}=t^{3}y_{1}-2ty_{2}-5t^{5}y_{3}-6t^{3}{\left(e^{t^{2}}y_{1}+t^{2}y_{2}+ty_{3}\right)}^{3}. \end{array}$$

**Figure 1:**
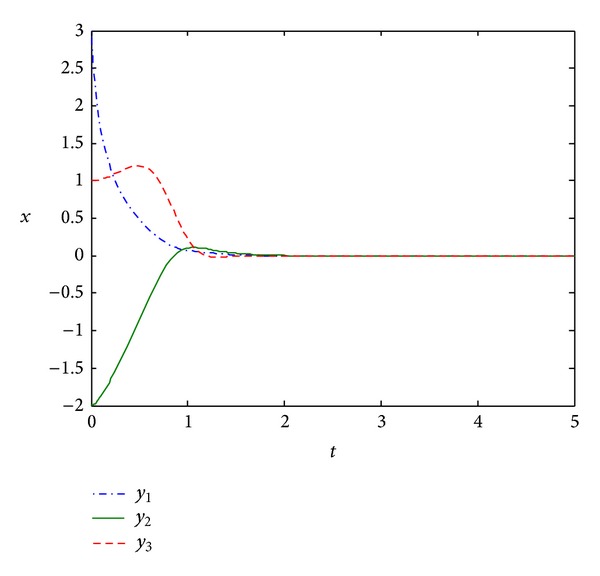
The state response of system (74).

Simulation results are shown in Figure 1. Although the coefficients of the large-scale Lurie direct control system with time-varying coefficients are norm-unbounded, we can clearly see from Figure 1 that the convergence rate of the system is very fast. This illustrates the availability of our results.

**Example 2**

Consider system (39) with

(75) $$\begin{array}{c} A_{11}\left(t\right)=\left(\begin{array}{cc} -2t & 1\\ t & -3t \end{array}\right),\;\;A_{12}\left(t\right)=\left(\begin{array}{c} 1\\ t^{2} \end{array}\right),\\ A_{21}\left(t\right)=\left(\begin{array}{cc} t^{3} & -2t \end{array}\right),\;\;A_{22}\left(t\right)=-5t^{5},\\ b_{11}\left(t\right)=\left(\begin{array}{c} -e^{t^{2}}\\ 2t \end{array}\right),\;\;b_{12}\left(t\right)=\left(\begin{array}{c} -e^{t^{2}}\\ 0 \end{array}\right),\\ b_{21}\left(t\right)=-6t^{3},\;\;b_{22}\left(t\right)=t^{2},\\ c_{11}\left(t\right)=\left(\begin{array}{c} e^{t^{2}}\\ t^{2} \end{array}\right),\;\;c_{12}\left(t\right)=t,\\ c_{21}\left(t\right)=\left(\begin{array}{c} e^{t^{2}}\\ 0 \end{array}\right),\;\;c_{22}\left(t\right)=2t^{2}. \end{array}$$

Similarly, we choose

(76) $$\begin{array}{c} T=1,\;\;P_{1}=\left(\begin{array}{cc} 1 & 0\\ 0 & 1 \end{array}\right),\;\;P_{2}=1. \end{array}$$

Then,

(77) $$\begin{array}{c} s_{1}\left(t\right)=2t,\;\;s_{2}\left(t\right)=10t^{5},\\ g_{1}\left(t\right)=e^{2t^{2}}-2t^{3},\;\;g_{2}\left(t\right)=e^{2t^{2}}-2t^{4}. \end{array}$$

This implies that assumptions A5 and A6 are satisfied. By calculating the upper limits, we have

(78) $$\begin{array}{c} \underset{t\to +\infty }{\bar{\lim}}\frac{2||P_{1}b_{11}\left(t\right)||}{\sqrt{s_{1}\left(t\right)g_{1}\left(t\right)}}={\bar{\alpha }}_{11}=0,\\ \underset{t\to +\infty }{\bar{\lim}}\frac{2||P_{1}b_{12}\left(t\right)||}{\sqrt{s_{1}\left(t\right)g_{2}\left(t\right)}}={\bar{\alpha }}_{12}=0,\\ \underset{t\to +\infty }{\bar{\lim}}\frac{2||P_{2}b_{21}\left(t\right)||}{\sqrt{s_{2}\left(t\right)g_{1}\left(t\right)}}={\bar{\alpha }}_{21}=0,\\ \underset{t\to +\infty }{\bar{\lim}}\frac{2||P_{2}b_{22}\left(t\right)||}{\sqrt{s_{2}\left(t\right)g_{2}\left(t\right)}}={\bar{\alpha }}_{22}=0,\\ \underset{t\to +\infty }{\bar{\lim}}\frac{||\sum_{j=1}^{2}c_{1j}^{T}\left(t\right)A_{j1}\left(t\right)+{\dot{c}}_{11}^{T}\left(t\right)||}{\sqrt{s_{1}\left(t\right)g_{1}\left(t\right)}}={\bar{\beta }}_{11}=0,\\ \underset{t\to +\infty }{\bar{\lim}}\frac{||\sum_{j=1}^{2}c_{1j}^{T}\left(t\right)A_{j2}\left(t\right)+{\dot{c}}_{12}^{T}\left(t\right)||}{\sqrt{s_{2}\left(t\right)g_{1}\left(t\right)}}={\bar{\beta }}_{12}=0,\\ \underset{t\to +\infty }{\bar{\lim}}\frac{||\sum_{j=1}^{2}c_{2j}^{T}\left(t\right)A_{j1}\left(t\right)+{\dot{c}}_{21}^{T}\left(t\right)||}{\sqrt{s_{1}\left(t\right)g_{2}\left(t\right)}}={\bar{\beta }}_{21}=0,\\ \underset{t\to +\infty }{\bar{\lim}}\frac{||\sum_{j=1}^{2}c_{1j}^{T}\left(t\right)A_{j2}\left(t\right)+{\dot{c}}_{12}^{T}\left(t\right)||}{\sqrt{s_{2}\left(t\right)g_{1}\left(t\right)}}={\bar{\beta }}_{22}=0. \end{array}$$

So, assumption A7′ is satisfied. From

(79) $$\begin{array}{c} \underset{t\to +\infty }{\bar{\lim}}\frac{2||P_{1}A_{12}\left(t\right)||}{\sqrt{s_{1}\left(t\right)s_{2}\left(t\right)}}={\bar{\gamma }}_{12}=0,\\ \underset{t\to +\infty }{\bar{\lim}}\frac{2||P_{2}A_{21}\left(t\right)||}{\sqrt{s_{2}\left(t\right)s_{1}\left(t\right)}}={\bar{\gamma }}_{21}=\frac{\sqrt{2}}{\sqrt{5}},\\ \underset{t\to +\infty }{\bar{\lim}}\frac{\left|\sum_{j=1}^{2}c_{1j}^{T}\left(t\right)b_{j2}\left(t\right)\right|}{\sqrt{g_{1}\left(t\right)g_{2}\left(t\right)}}={\bar{\mu }}_{12}=0,\\ \underset{t\to +\infty }{\bar{\lim}}\frac{\left|\sum_{j=1}^{2}c_{2j}^{T}\left(t\right)b_{j1}\left(t\right)\right|}{\sqrt{g_{2}\left(t\right)g_{1}\left(t\right)}}={\bar{\mu }}_{12}=0, \end{array}$$

we know that assumptions A8′ and A9′ are satisfied and

(80) Q¯=(−1γ¯12α¯11α¯12γ¯21−1α¯21α¯22β¯11β¯12−1μ¯12β¯21β¯22μ¯21−1)=(−100025−10000−10000−1).

It is easy to see that $\;\bar{Q}\;$ is stable. Hence, according to Corollary 11, the system is absolutely stable.

Let *f*_1_(*σ*_1_) = *σ*_1_^3^, *f*_2_(*σ*_2_) = *σ*_2_^5^, and$\;x_{1}=\left(\begin{array}{c} y_{1}\\ y_{2} \end{array}\right),\;x_{2}=y_{3}$; then system (39) can be written as

**Figure 2:**
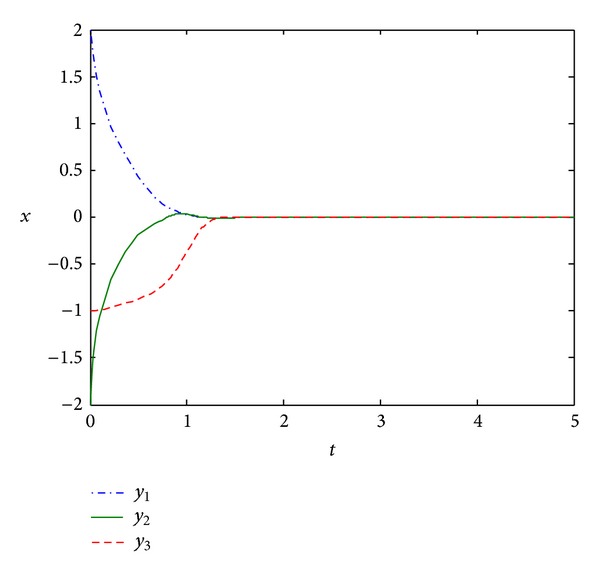
The state response of system (81).

(81) y˙1=−2ty1+y2+y3−et2(et2y1+t2y2+ty3)3−et2(et2y1+2t2y3)5,y˙2=ty1−3ty2+t2y3+2t(et2y1+t2y2+ty3)3,y˙3=t3y1−2ty2−5t5y3−6t3(et2y1+t2y2+ty3)3+t2(et2y1+2t2y3)5,

as shown in Figure 2. For the large-scale Lurie direct control system with time-varying coefficients and multiple nonlinearities, although the coefficients are norm-unbounded, we can see from Figure 2 that the large-scale system is absolutely stable.

## 5. Conclusions

The absolute stability of large-scale Lurie direct control systems with time-varying coefficients and systems with multiple nonlinearities is studied in this paper. By restricting the relative magnitude of the time-varying coefficients and employing the decomposition theory of large-scale systems, some absolute stability criteria were obtained. The criteria, introduced in this paper, can be used not only in large-scale Lurie direct control systems with norm-unbounded coefficients but also in systems with norm-bounded coefficients. Two numerical examples are introduced to illustrate the availability of our results.
